# A comparison of opioid dose between home palliative care and hospital palliative care

**DOI:** 10.1186/s12875-024-02265-z

**Published:** 2024-01-23

**Authors:** Hiroyoshi Iwata, Shuhei Hamada, Hiroko Harada, Katsuhiro Kusaka

**Affiliations:** 1 Center for Environmental and Health Sciences, Hokkaido University, North-12, West-7, Kita-Ku, Sapporo, Hokkaido, 060-0812 Japan; 2 Department of Home Care Medicine, Ebetsu Visiting Home Care Clinic, Hokkaido, Japan; 3 Department of Primary Care and Medical Education, Institute of Medicine, University of Tsukuba, Ibaraki, Japan; 4 Department of Internal Medicine, Kamisu Saiseikai Hospital, Ibaraki, Japan; 5 Department of Palliative Internal Medicine, Sapporo City General Hospital, Hokkaido, Japan

**Keywords:** Palliative care, Home palliative care, Opoid

## Abstract

**Objective:**

While opioids are a key part of palliative care, few studies have evaluated opioid demand in the home care context. This study aims to compare opioid usage in home care and hospital care settings.

**Methods:**

This cross-sectional study retrospectively recruited patients receiving palliative care in home care and hospital settings, between November 2018 and October 2020. Opioid prescriptions were standardized to oral morphine equivalent (OME) doses at 7 and 14 days prior to death and analyzed. Additional analysis performed multivariable linear regression on the outcome of OME at 7 days, adjusting for medical setting and confounders in patients with opioid prescriptions.

**Results:**

After 21 exclusions, 209 patients (48 home care and 161 hospital care) were eligible for analysis. The home care group had a higher mean age (74.8 years) and Palliative Prognosis Score (50), than the hospital group (70.1 and 40, respectively). Mean OME at 7 and 14 days before death was numerically higher in the home care group (72.8 mg/day and 53.0 mg/day, respectively) than the hospital care group (57.7 mg/day and 35.7 mg/day). Student’s t-test produced *p*-values of 0.49 and 0.32, and the Wilcoxon rank sum test found *p*-values of 0.24 and 0.11 at 7 and 14 days, respectively. Multivariable regression analysis of the home care group found mean OME of 40.7 mg/day; 95% confidence interval [-0.62, 82.0 (mg/day)], *p* = 0.06. Additional analysis found a *p*-value of 0.06 for medical setting.

**Conclusions:**

We did not find a statistically significant difference in opioid use between home care and hospital care. However, the numerically higher rate of use in the home care group suggests that further research is warranted.

**Supplementary Information:**

The online version contains supplementary material available at 10.1186/s12875-024-02265-z.

## Introduction

The global trend toward aging populations and increasing rates of malignancy has brought growing attention to home palliative care. As of 2022, Japan has one of the world's most aged populations, and this super-aging society is expected to continue [1]. As a society ages, providing for wellbeing during old age and the final days of life becomes a pressing issue, and home care is an attractive option for responding to these needs. The Japanese Ministry of Health, Labour and Welfare has promoted a "Regional Medical Plan" to create a system that supports home care, including end-of-life care [2]. The Regional Medical Plan aims to facilitate home medical care, allowing patients to end their lives at home or in a nursing home, rather than in a hospital; in response to Japan’s growing elderly population, the plan is designed as a system in each prefecture to support coordination and enhancement of the numbers of medical facilities and staff in order to enable effective home care [2]. Treatment with opioids has been a key strategy in cancer pain management for over two centuries [3, 4]. As malignant tumors are now the leading cause of death among Japanese people (27.4% in 2018) [5], the demand for home palliative care among cancer patients can be expected to increase further, underscoring the importance of appropriate opioid use as an essential element of palliative care.

Despite the growing importance of home care, few studies have investigated the factors affecting pain control and opioid administration in that setting when compared with hospital care. A study of 303 patients by Jamison et al. found that patients with chronic pain who lacked family support experienced more intense pain and stress than those who received support from their families [6], suggesting that home care patients receiving family support may need fewer opioids. Similarly, Takizawa et al. reported that home care patients needed lower amounts of opioids than hospitalized patients [7]. Conversely, home care patients are not always located near their health care providers and may require more opioids in response to exacerbation of pain due to anxiety and other factors. The study by Takizawa et al. implies that the same clinicians provided care to both the home care group and the hospitalized group; because patients with more severe pain were considered for admission, their results may not accurately compare opioid demand among hospital and home care patients.

In order to compare opioid demand in home care and hospital care settings, we investigated opioid administration among patients receiving end-of-life care at home, managed by a home care support clinic, and patients admitted at a general hospital, managed by a palliative care medicine department.

## Methods

This multi-center retrospective cohort study followed the Strengthening the Reporting of Observational Studies in Epidemiology (STROBE) Statement [8] (File S1). We searched the medical records of consecutive patients who were followed by one home care clinic and consecutive patients admitted to one general hospital between November 1, 2018 and October 31, 2021.

### Inclusion and exclusion criteria

We recruited all eligible patients who received home or admitted palliative care at both institutions during the search period. Our study included cancer patients who died at one general hospital supported by its palliative care team (hospital care group) or who died at home supported by one home care clinic team (home care group). The hospital and clinic are located in neighboring cities. We excluded patients who 1) did not have malignant diseases, 2) received care in the intensive care unit (ICU), 3) received nerve blocks, 4) were transferred from home care for the purpose of pain control, 5) were discharged from the hospital to their homes, or 6) died suddenly during temporary discharge. We recruited eligible patients consecutively to minimize bias.

### Data collection

We collected patient data including sex, age, medical history, administered oxygen, Palliative Performance Scale (PPS), comorbidity and Charlson Comorbidity Index (CCI), primary disease, length of hospital stay or period of management by the clinic, symptoms, laboratory data such as albumin and estimated glomerular filtration rate, and palliative care drugs including opioid type and dose.

### Measurement of oral morphine equivalent opioid dose

Oral morphine equivalent (OME) opioid dose was calculated using data extracted from medical records. Because Japan has a legally mandated system for monitoring and reporting opioids which are prescribed but not actually consumed by patients, the medical records were deemed likely to contain accurate information on actual opioid administration. We evaluated OME as of 7 days before death, because physicians may decrease or halt opioid administration if a patient loses consciousness at the terminal stage. Conversion of doses of other opioids such as oxycodone, fentanyl, and methadone to OME was performed using the equivalency ratios presented in Table S1 [9–11, 12].

### Assessment of insufficient opioid administration

Although the Visual Analogue Scale (VAS) is ideal for assessing pain control, obtaining VAS information was impossible due to the retrospective nature of this study. Moreover, evaluation by physicians based solely on medical charts often lacks objectivity. The Japanese Clinical Guidelines for Cancer Pain Management (2014) recommend increasing VAS evaluations if rescue use of opioids occurs four or more times in a day [13]. Therefore, although not an established method, we defined poor pain control as rescue use four or more times per day, during the period from 14 to 7 days prior to death.

### Data analysis

Differences in clinical characteristics were analyzed using descriptive statistics: median, first quartile and third quartile for continuous variables, and percentages for categorical variables between the patients in the hospital care group and those in the home care group. We compared the hospital and home care groups using Student's t test for continuous variables, and the chi-square test or Fisher’s exact test when over 20% of cells had expected frequencies < 5 for categorical variables. Furthermore, in order to compare the OME dosage among the patients who needed opioids, we created a summary table and box plots comparing home care and hospital care. For statistical analysis of differences in OME dosage, we used Student’s t-test. Given the possibility that the data are not normally distributed, we also conducted the Wilcoxon rank sum test. We used two-tailed tests and defined statistical significance as *p* < 0.05. As a sensitivity analysis, we conducted Student’s t-test and the Wilcoxson rank sum test again using all participants before exclusion.

Additionally, in order to assess dosing among the patients who received opioid prescriptions, we performed a multivariable linear regression analysis employing models using OME dose as the outcome, adjusting for care setting (home care or hospital) and clinical confounders (patient age, PPS, cancer type, and pain control). Confounders were chosen based on clinical expert inspections and the number of participants. We evaluated the residuals to assess the validity of our linear regression using a QQ plot generated with the native function “qqnorm” in R (version 4.0.3; R Foundation for Statistical Computing, Vienna, Austria). We then used the “qqPlot” function in the “car” package to identify any outliers, and conducted the regression analysis again after removing the outliers [14]. We omitted missing data when conducting statistical analysis. Statistical analysis was performed using R (version 4.0.3; R Foundation for Statistical Computing, Vienna, Austria) and Stata Statistical Software (Release 15; StataCorp LLC, College Station, TX).

Sample sizes were calculated based on the Wilcoxon rank sum test, a nonparametric method, assuming that OME dosage data would not be normally distributed. The R package "samplesize" was used for this calculation [15]. OME dose was stratified into three groups (0–100 mg, 101–200 mg, and 201 mg or more). The sample size calculation required a total of 221 patients (55 in the home care group and 166 in the hospital care group).

### Ethical considerations

This study was approved by the ethics committees at the authors’ institutions. The patient consent form used an opt-out method. Eligible patients received explanations about the study and notification that they were able to opt out. This research was conducted in accordance with the "Declaration of Helsinki (amended in October 2013)" and the "Ethical Guidelines for Medical and Health Research Involving Human Subjects" in Japan.

## Results

**Fig. 1:**
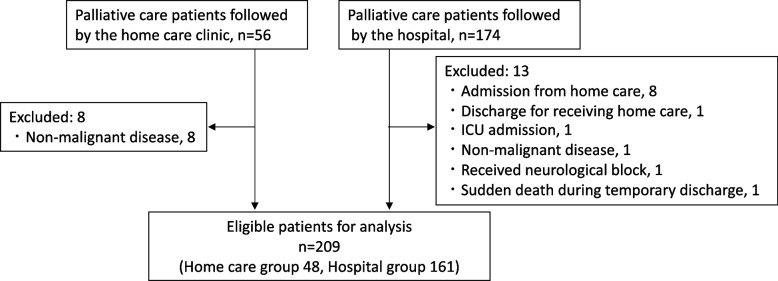
Study flow chart for evaluation of opioid dosage among home care and hospital care patients

In the present cohort study, 230 candidates were identified from among deceased adult patients managed by one general hospital and one home care clinic, using the medical flow charts at both institutions. We excluded 21 patients; 9 with non-malignant disease, 8 who were transferred from home care to hospital for better pain control, 1 who received nerve block, 1 admitted to the ICU, 1 who died suddenly during temporary discharge, and 1 who was discharged from the hospital to receive care at home. As a result, 209 patients (48 home care patients and 161 hospital care patients) were eligible for analysis (Fig. 1).

Patient characteristics are summarized in Table 1. The mean age of the home care group was about 5 years older than the hospital care group. The hospital care group scored lower on the PPS and higher on the CCI, compared with the home care group. There were no obvious differences in sex. At 7 and 14 days prior to death, the proportion of patients with consciousness disturbance was statistically significantly higher in the home care group than in the hospital care group. Median PPS scores in the hospital care and the home care groups were 40.0 and 50.0 respectively. Significant differences were also found in the statistical distributions of age, serum albumin, and follow-up period, as well as dyspnea, non-steroidal anti-inflammatory drug (NSAID) usage, and anti-psychotic drug usage at 14 days prior to death, and NSAID usage at 7 days prior to death (Table 1).

**Table 1:** Basic characteristics and opioid dosage, palliative care drug usage of the home care group and the hospital care group

	**Hospital group**	**Home care group**	**Overall**	*P*-value
	**(*n* = 161)**	**(*n* = 48)**	**(*n* = 209)**	
**Age**				0.02^*^
Mean (SD)	70.1 (12.3)	74.8 (12.1)	71.2 (12.4)	
Median [Q1, Q3]	72.0 [64.0, 79.0]	76.0 [66.8, 84.8]	72.0 [64.0, 79.0]	
**Sex**				0.70^**^
Female	80 (49.7%)	26 (54.2%)	106 (50.7%)	
Male	81 (50.3%)	22 (45.8%)	103 (49.3%)	
**Serum Alb, ng/dl**				0.01^*^
Mean (SD)	2.32 (0.674)	2.60 (0.611)	2.38 (0.670)	
Median [Q1, Q3]	2.35 [1.80, 2.70]	2.60 [2.10, 3.00]	2.40 [1.90, 2.80]	
Missing				
**Charlson Comorbidity Index**				0.50^*^
Mean (SD)	6.65 (2.52)	6.38 (2.18)	6.58 (2.44)	
Median [Q1, Q3]	7.00 [6.00, 8.00]	6.00 [6.00, 8.00]	7.00 [6.00, 8.00]	
**Palliative Prognosis Score**				0.32^*^
Median [Q1, Q3]	40.0 [30.0, 50.0]	50.0 [30.0, 50.0]	40.0 [30.0, 50.0]	
**Follow up period days**				< 0.001^*^
Median [Q1, Q3]	20.0 [10.0, 32.0]	40.0 [21.0, 66.0]	22.0 [12.0, 41.0]	
**Conscinousness disturbance 14 days before death, %**				< 0.001^**^
Positive	15 (9.3%)	13 (27.1%)	28 (13.4%)	
Negative	146 (90.7%)	35 (72.9%)	181 (86.6%)	
**Dyspnea 14 days before death, %**				0.01^†^
Positive	27 (16.8%)	1 (2.1%)	28 (13.4%)	
Negative	134 (83.2%)	47 (97.9%)	181 (86.6%)	
**Conscinousness disturbance 7 days before death, %**				< 0.001^**^
Positive	21 (13.0%)	22 (45.8%)	43 (20.6%)	
Negative	140 (87.0%)	26 (54.2%)	166 (79.4%)	
**Dyspnea 7 days before death, %**				0.31^†^
Positive	36 (22.4%)	7 (14.6%)	43 (20.6%)	
Negative	125 (77.6%)	41 (85.4%)	166 (79.4%)	
**Maligncy types**				0.39^†^
Blood	12 (7.5%)	3 (6.3%)	15 (7.2%)	
Breast	8 (5.0%)	2 (4.2%)	10 (4.8%)	
Gastrointestinal	63 (39.1%)	26 (54.2%)	89 (42.6%)	
Gynecology	4 (2.5%)	5 (10.4%)	9 (4.3%)	
Lung	39 (24.2%)	8 (16.7%)	47 (22.5%)	
Urology	19 (11.8%)	2 (4.2%)	21 (10.0%)	
Others	16 (9.9%)	2 (4.2%)	18 (8.6%)	
**Oral morphine equivalent dose 14 days before death, mg/day**				0.32^*^
Mean (SD)	35.7 (102)	53.0 (116)	39.7 (105)	
Median [Q1, Q3]	0 [0, 30.0]	15.0 [0, 40.0]	0 [0, 30.0]	
**Non-steroidal anti-inflammatory drug usage 14 days before death, %**				< 0.001^**^
User	29 (18.0%)	21 (43.8%)	50 (23.9%)	
Non-user	132 (82.0%)	27 (56.3%)	159 (76.1%)	
**Anti-psychotic drug usage 14 days before death, %**				0.01^†^
User	3 (1.9%)	6 (12.5%)	9 (4.3%)	
Non-user	158 (98.1%)	42 (87.5%)	200 (95.7%)	
**Continous intravnous sedation usage 14 days before death, %**				0.32†
User	3 (1.9%)	2 (4.2%)	5 (2.4%)	
Non-user	158 (98.1%)	46 (95.8%)	204 (97.6%)	
**Oral morphine equivalent dose 7 days before death, mg/day**				0.49^*^
Mean (SD)	57.7 (131)	72.8 (142)	61.2 (133)	
Median [Q1, Q3]	15.0 [0, 56.0]	23.3 [0, 78.8]	16.0 [0, 60.0]	
**Non-steroidal anti-inflammatory drug usage 7 days before death, %**				0.01^**^
User	32 (19.9%)	18 (37.5%)	50 (23.9%)	
Non-user	129 (80.1%)	30 (62.5%)	159 (76.1%)	
**Anti-psychotic drug usage 7 days before death, %**				0.07^†^
User	5 (3.1%)	7 (14.6%)	12 (5.7%)	
Non-user	156 (96.9%)	41 (85.4%)	197 (94.3%)	
**Continous intravnous sedation usage 7 days before death, %**				0.66^†^
User	5 (3.1%)	2 (4.2%)	7 (3.3%)	
Non-user	156 (96.9%)	46 (95.8%)	202 (96.7%)	
**Pain control failure between 7 and 14 days before death**				1.00^†^
Positive	10 (6.2%)	3 (6.3%)	13 (6.2%)	
Negative	151 (93.8%)	45 (93.8%)	196 (93.8%)	

^*^Student’s t test; comparing hospital group and home care group

^**^Chi-square test; comparing hospital group and home care group

^†^Fisher’s exact test; comparing hospital group and home care group

Opioid dose and usage of other palliative care drugs are summarized in Table 2. Comparisons of OME dosage between the home care and hospital care settings among patients who needed opioids are presented in Table 2. These results suggest that patients in the home care group received numerically higher daily opioid doses than those in the hospital care group, though this difference did not reach statistical significance. The proportion of patients who needed NSAIDs, anti-psychotic drugs, and intravenous sedatives was higher in the home care group than in the hospital care group at 7 and 14 days before their deaths. Irrespective of opioid dosage and frequency of rescue use, 3 and 10 participants in the home and hospital care groups, respectively, are suspected to have been suffering from insufficient pain control during the period between 7 and 14 days before their deaths.

**Table 2:** Opioid dosage, palliative care drug usage among patients receiving opioid prescriptions in the home care group and hospital care group

Setting	Participants without opioid prescriptions, n (%)^a^	Participants with opioid prescriptions, n (%)^a^	Mean^b^	Minimum^b^	First Quartile^b^	Median^b^	Third Quartile^b^	Maximum^b^
Hospital care	60 (37.3%)	101 (62.7%)	92	2.5	20	40	90	960
Home care	14 (29.2%)	34 (70.8%)	103	12	19	48	95	840

^a^The timing of evaluation was 7 days prior to death

^b^Oral morphine equivalent dose (mg/day)

Student’s t-test did not detect a statistically significant difference between the home care group and the hospital care group in OME dose per day, with *p*-values of 0.493 and 0.319 at 7 and 14 days before death, respectively. Wilcoxon rank sum test results similarly failed to detect any statistically significant difference between the home care group and the hospital care group in OME dose per day at 7 and 14 days before death, *p* = 0.243 and 0.111, respectively. Sensitivity analysis involving all 230 patients before exclusion (56 in the home care group and 174 in the hospital care group) produced *p*-values of 0.662 and 0.536 with Student’s t-test and 0.338 and 0.164 with the Wilcoxon rank sum test, respectively.

Results from our additional analysis are provided in Table 3. The multivariable linear regression analysis of care settings found a numerical difference of 40.7 OME between the home care clinic and the hospital, but this did not reach statistical significance; the 95% Confidence interval was from -0.62 to 82.0 (*p* = 0.055), among the patients who needed opioids. The QQ plots show that the corresponding distributions are fitted in a line (Figure S1). Because the QQ plot and analysis using “qqPlot” identified two right edge outliers, we conducted univariable and multivariable regressions after removing them, and confirmed the same trend found in the main analysis. Thus, multivariable regression also did not find a statistically significant difference in opioid administration between the home care and hospital care groups.

**Table 3:** Multivariable linear regression results adjusting for medical setting and confounders

Variable	β-coefficient	Standard Error	Lower 95% CI^a^	Upper 95% CI^a^	*P*-value
Setting	40.69	21.08	-0.62	81.99	0.055
Age	-3.89	0.74	-5.33	-2.45	0.000
Pain control failure	90.45	35.99	19.90	160.99	0.013
Palliative Performance Scale	-1.14	0.47	-2.06	-0.22	0.016
Breast	-72.88	50.27	-171.42	25.65	0.149
Gastrointestinal	-3.76	34.72	-71.81	64.30	0.914
Gynecology	-20.40	52.19	-122.69	81.88	0.696
Lung	-2.56	36.99	-75.05	69.93	0.945
Urology	16.84	41.69	-64.87	98.55	0.687
Others	24.82	42.93	-59.32	108.96	0.564

^a^*CI* Confidence interval

## Discussion

The present study did not find a statistically significant difference in opioid needs between home care and hospitalized patients, though the home care group had a numerically higher rate of opioid use, suggesting that home care patients may need more opioids. These results highlight the demand for opioid treatment within the context of home care. Future large-scale, multi-center studies are needed to further elucidate the role of opioid therapy in home palliative care.

Demographic differences between the groups may explain the lack of significant findings in this study. Importantly, both the average and median ages were higher in the home care group than in the hospital care group. In general, younger patients tend to require more opioids in palliative care [16]. Therefore, the hospital care group in this study could be expected to have a higher opioid usage rate. However, patients in the home care group required similar or potentially greater amounts of opioids for pain management when compared to the hospital care group, suggesting the possibility that home-care patients need more opioids than hospital-care patients. Moreover, while our regression results adjusted for confounders including age are not statistically significant, the *p*-values are lower than in the primary analysis and approach 0.05, which may suggest that a significant difference could be found in a larger study.

Several potential reasons might explain why home care patients may need more opioids than patients receiving hospital care. In the home care setting, medical staff are not always available, leading to longer intervals between visits and less frequent adjustment of medications. Additionally, physical isolation from medical staff might lead to increased psychological stress and worry, thereby exacerbating pain. In the present study, the higher prescription rates of NSAIDs and anti-psychotic drugs in the home care group suggests that their clinicians were attentive not only to pain management but also to their psychiatric symptoms, potentially in response to input from family members. Furthermore, home care patients may be actively prescribed opioids in response to expressions of concern from family members. Conversely, family members typically have fewer opportunities to visit and interact with hospital inpatients, meaning that they are less likely to request health care providers to provide opioid pain treatment.

Our study suggests novel clinical concerns related to home palliative care. It has been suggested previously that opioid needs are lower in the home than in the hospital [7]. However, our results suggest that home care patients do not have reduced needs for opioid pain management, and may even need more than patients in hospital care. Clinicians must carefully consider how to provide sufficient opioid pain relief to home care patients. In response to growing concerns about the crisis of opioid overuse, prescriptions to patients suffering from poor prognosis cancers declined substantially from 2007 to 2017 in the US [17]. Potential overuse of opioids among terminally ill outpatients, though not specifically in the context of home care, has also been cited as a challenge in France [18]. The same trend may hold true in the context of home care, meaning that these patients may be suffering from insufficient pain management due to their unique needs. Clinicians providing palliative care to patients in their homes must therefore balance a range of considerations in order to safely provide adequate opioid-based pain relief.

Although this study did not find any significant differences, its design strengths support the reliability of the results. First, in order to avoid potential difficulties in assessing proper opioid usage near the end of life, due to factors such as impaired consciousness and sedation, we assessed OME dosage at two points, 7 and 14 days before death. Our sample size was set to allow for three OME categories, considering that OME dosage may not have a normal distribution due to a small minority of patients receiving an outsized share of opioid doses in response to severe pain. Next, we conducted the present study in two medical institutions located in adjacent cities, and recruited patients consecutively in order to minimize selection bias. Finally, we evaluated pain control failure.

Our study did not identify a statistically significant difference in the amount of opioids prescribed between home-based and hospital-based medical care. This leaves open the possibility that the two are equivalent, though our study did not include equivalence tests. If there is no disparity in opioid usage between hospital and home settings, a greater number of terminally-ill cancer patients may feel comfortable choosing home care. This would not only help address the needs of patients who wish to spend their final moments at home, but also potentially contribute to reductions in overall nationwide healthcare costs.

Our study also has some important limitations. First, there are inherent challenges in gathering information on patients receiving care at home and in hospital in order to compare the two settings. For example, data needed to compute BMI are difficult to measure at home, while there are difficulties in obtaining the number of family members of inpatients in the hospital setting. We performed regression analysis with patient age, PPS, cancer type and pain control as confounders, but were unable to perform an analysis that fully incorporated all potential confounders. Second, the inclusion of data from only two institutions raises concerns about external validity and the possibility of bias. Because there are very few pertinent studies in the field of palliative home care, it was difficult to estimate in advance how opioid use in home care would compare to the hospital context. However, we recruited patients consecutively to minimize bias. Further, due to the two-center design and limited sample size available for this study, we could not employ more sophisticated statistical methods such as multilevel analysis, so we decided to focus on basic and descriptive statistics. Additionally, some opioids appearing in our study, such as methadone, are difficult to convert to OME, which may influence the accuracy of our findings. Thirdly, while the present study was conducted at two medical centers, its relatively small scope may limit the external validity of our findings. A further multicenter study is therefore warranted. Finally, although we performed preliminary sample size calculations to allow for the detection of potentially significant differences, the OME data that we gathered had an unexpectedly wide variance, which limited our ability to detect differences.

## Conclusion

Although the results of the present study failed to reach statistical significance, they suggest the possibility that patients receiving home palliative care may have greater demand for opioids than hospitalized patients. Our results differ from those of the few available previous studies. Accordingly, larger multicenter studies are warranted to investigate the need for opioids in this clinically important group.

### Supplementary Information

**Additional file 1:** **File S1.** STROBE Statement—checklist of items that should be included in reports of observational studies.

**Additional file 2:** **Table S1.** Daily total oral morphine dose equivalency ratios, mg.

**Additional file 3:** **Figure S1.** QQ Plot Results.

## Data Availability

Aggregated data used in this study is available upon request.

## References

[1] World Population Ageing, United Nations (2017),http://www.un.org/en/development/desa/population/publications/pdf/ageing/WPA2017_Highlights.pdf. Accessed 10 Aug 2020.

[2] Community-oriented primary care, Ministry of Health, Labour and Welfare, Japanese Government. URL: https://www.mhlw.go.jp/stf/seisakunitsuite/bunya/0000080850.html. Accessed 3 Jan 2023.

[3] Bruera E, Paice JA. Cancer pain management: safe and effective use of opioids. Am Soc Clin Oncol Educ Book. 2015;35:e593–9. 10.14694/EdBook_AM.2015.35.e593 25993228

[4] Kurita GP, Kaasa S, Sjøgren P, Bruera E, Higginson I, von Gunten C (2015). Opioid Analgesics. Textbook of Palliative Medicine and Supportive Care.

[5] Death causes by sex, Vital statistics (2018), Ministry of Health, Labour and Welfare, Japanese Government. URL: https://www.mhlw.go.jp/toukei/saikin/hw/jinkou/kakutei18/dl/10_h6.pdf. Accessed 3 Jan 2023.

[6] Jamison RN, Virts KL (1990). The influence of family support on chronic pain. Behav Res Ther.

[7] Takizawa Y, Shimodeda S, Nishizawa S (2010). Current State and Problems of Opioid Administration in Terminal-Stage Cancer Patients Treated at Home. Jpn J Pharm Palliat Care Sci..

[8] von Elm E, Altman DG, Egger M, Pocock SJ, Gøtzsche PC, Vandenbroucke JP (2007). The Strengthening the Reporting of Observational Studies in Epidemiology (STROBE) statement: guidelines for reporting observational studies. Ann Intern Med.

[9] Group WSAMD. AMDG 2015 interagency guideline on prescribing opioids for pain. Washington State Agency Medical Directors’ Group Olympia, WA; 2015. URL: https://www.agencymeddirectors.wa.gov/Files/2015AMDGOpioidGuideline.pdf. Accessed 3 Jan 2023.

[10] G. AMDs, “Opioid Dose Calculator,” 2020, URL: https://amdg.wa.gov/calculator/DoseCalculator. Accessed 3 Jan 2023.

[11] Broglio, Post. FURL: https://www.uptodate.com/contents/image?imageKey=PALC%2F111216. Accessed 3 Jan 2023.

[12] National Cancer Center Japan, Opioid drug conversion table. 2019. Available: https://www.ncc.go.jp/jp/ncch/clinic/palliative_care/201901opioid.pdf.

[13] Japanese Society for Palliative Medicine. Clinical guidelines for cancer pain management, 2nd edition. Kanehara & Co., Ltd. Tokyo, Japan; 2014. Available: https://www.jspm.ne.jp/files/guideline/pain_2014/pain2014.pdf.

[14] Fox J, Weisberg S (2019). An R Companion to Applied Regression, Third edition. Sage, Thousand Oaks CA. https://socialsciences.mcmaster.ca/jfox/Books/Companion/.

[15] Scherer, R. (2016) samplesize 0.2–4: Sample size calculation for various t-tests and Wilcoxon-test. https://CRAN.R-project.org/package=samplesize. Accessed 3 Jan 2023.

[16] Yamashita K, Nabeshima A, Hara Y, Okochi J (2007). Influence of body weight, age, and primary tumor site on opioid dose in advanced cancer pain patients. Nihon Ronen Igakkai Zasshi.

[17] Enzinger AC, Ghosh K, Keating NL, Cutler DM, Landrum MB, Wright AA (2021). US Trends in Opioid Access Among Patients With Poor Prognosis Cancer Near the End-of-Life. J Clin Oncol.

[18] Chu TH, Rueter M, Palmaro A, Lapeyre-Mestre M (2022). Potential inappropriate use of strong opioid analgesics in cancer outpatients during the last year of life in France and associated factors. Br J Clin Pharmacol.

