# Supplementary figures and images for: A comparison of opioid dose between home palliative care and hospital palliative care
Source: BMC Prim Care. 2024 Jan 23;25:33. doi: 10.1186/s12875-024-02265-z (PMC10804711; doi:10.1186/s12875-024-02265-z)

Table S1. Daily total oral morphine dose equivalency ratios, mg


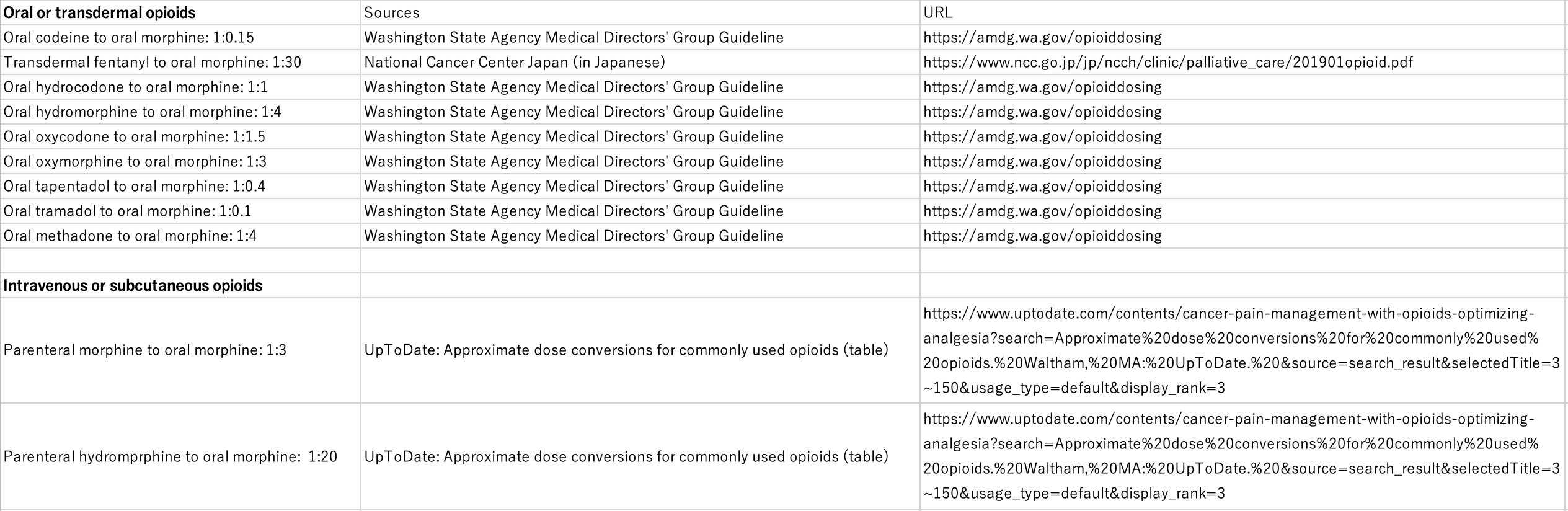

Supplement: Supplementary file 2 — Additional file 2: Table S1. Daily total oral morphine dose equivalency ratios, mg. [file 12875_2024_2265_MOESM2_ESM.docx]
