# Supplementary material for: A comparison of opioid dose between home palliative care and hospital palliative care
Source: BMC Prim Care. 2024 Jan 23;25:33. doi: 10.1186/s12875-024-02265-z (PMC10804711; doi:10.1186/s12875-024-02265-z)
Supplement: Supplementary file 3 — Additional file 3: Figure S1. QQ Plot Results. [file 12875_2024_2265_MOESM3_ESM.docx]

Figure S1. QQ Plot Results

A: QQ plot with univariable regression.

*identified as outliers.

B: QQ plot with multivariable regression

*identified as outliers.
